# Developmental Changes in the Metabolic Network of Snapdragon Flowers

**DOI:** 10.1371/journal.pone.0040381

**Published:** 2012-07-11

**Authors:** Joëlle K. Muhlemann, Hiroshi Maeda, Ching-Yun Chang, Phillip San Miguel, Ivan Baxter, Bruce Cooper, M. Ann Perera, Basil J. Nikolau, Olga Vitek, John A. Morgan, Natalia Dudareva

**Affiliations:** 1 Department of Horticulture and Landscape Architecture, Purdue University, West Lafayette, Indiana, United States of America; 2 Department of Statistics, Purdue University, West Lafayette, Indiana, United States of America; 3 Bindley Bioscience Center, Metabolite Profiling Facility, Purdue University, West Lafayette, Indiana, United States of America; 4 W.M. Keck Metabolomics Research Laboratory, Iowa State University, Ames, Iowa, United States of America; 5 Department of Biochemistry, Biophysics, and Molecular Biology, Iowa State University, Ames, Iowa, United States of America; 6 School of Chemical Engineering, Purdue University, West Lafayette, Indiana, United States of America; Max Planck Institute for Chemical Ecology, Germany

## Abstract

Evolutionary and reproductive success of angiosperms, the most diverse group of land plants, relies on visual and olfactory cues for pollinator attraction. Previous work has focused on elucidating the developmental regulation of pathways leading to the formation of pollinator-attracting secondary metabolites such as scent compounds and flower pigments. However, to date little is known about how flowers control their entire metabolic network to achieve the highly regulated production of metabolites attracting pollinators. Integrative analysis of transcripts and metabolites in snapdragon sepals and petals over flower development performed in this study revealed a profound developmental remodeling of gene expression and metabolite profiles in petals, but not in sepals. Genes up-regulated during petal development were enriched in functions related to secondary metabolism, fatty acid catabolism, and amino acid transport, whereas down-regulated genes were enriched in processes involved in cell growth, cell wall formation, and fatty acid biosynthesis. The levels of transcripts and metabolites in pathways leading to scent formation were coordinately up-regulated during petal development, implying transcriptional induction of metabolic pathways preceding scent formation. Developmental gene expression patterns in the pathways involved in scent production were different from those of glycolysis and the pentose phosphate pathway, highlighting distinct developmental regulation of secondary metabolism and primary metabolic pathways feeding into it.

## Introduction

Flowers are amongst the most important plant organs as they are vital for the plant’s reproductive and evolutionary success. Flower development requires a tight spatial and temporal regulation of cellular processes and is accompanied by changes in cellular metabolism. It is initiated by the induction of a flower primordium [1], followed by subsequent cell divisions and expansion, which govern growth and final shape of the organ [2]. Reproductive maturity of the flower is reached when the stigma and anthers are ready for pollination. Senescence terminates the lifespan of the flower and is typically triggered by pollination or signaling mechanisms inducing programmed cell death [3]. To ensure successful pollination and completion of the plant’s life cycle, diverse angiosperm flowers have evolved sophisticated visual and olfactory cues that attract pollinators to the reproductive organ. Visual signaling in flowers involves the production of pigments such as anthocyanins, carotenoids, and betalains [4], [5], [6]. Olfactory cues are generated by the emission of a diverse blend of volatile organic compounds synthesized by various biosynthetic pathways including the phenylpropanoid and terpenoid pathways [7]. Pigment and volatile production are developmentally regulated and temporally separated. Biosynthesis of pigments generally begins at early stages of development and continues until the petals are fully expanded [4], [5], [6]. In contrast, the biosynthesis of volatiles usually begins only after anthesis (flower opening) and reaches its maximum when flowers are ready for pollination [8], [9], [10], [11].

Flowers predominantly rely on a supply of sucrose - the major photosynthate translocated from source to sink tissues - for the generation of energy, macromolecules, and organic compounds involved in primary and secondary plant metabolism [12]. To date, analysis of the developmental regulation of flower metabolism has predominantly focused on the biosynthesis of pigments and volatile scent compounds, as well as carbohydrate metabolism involved in flower opening [4], [5], [6], [7], [13]. However, how the entire metabolic network – from sucrose to secondary metabolites – operates during flower development has yet to be determined. To gain a comprehensive understanding of metabolic changes occurring over flower development, we analyzed the transcriptome and metabolome of non-photosynthetic petals and photosynthetically active sepals over flower development using *Antirrhinum majus* (snapdragon) flowers. Snapdragon is a well-characterized model system for the investigation of floral development [1], [2], pigment production [5], and scent emission [8], [9], [10], [11]. Snapdragon flowers emit a blend of phenylpropanoid and terpenoid compounds, which is dominated by the three monoterpenes myrcene, β-ocimene, and linalool, the sesquiterpene (E)-nerolidol, and the phenylpropanoid methylbenzoate [8], [9], [10], [11]. Emission of these compounds is developmentally regulated and follows a diurnal rhythm that is controlled by a circadian clock [14]. These volatiles are synthesized in the epidermis of upper and lower petal lobes [15], where anthocyanin pigments also accumulate [5]. Genes controlling snapdragon flower development [16], [17], as well as genes encoding enzymes involved in anthocyanin and volatile biosynthesis have been isolated and characterized in recent years [8], [9], [10], [11], [17]. It has been shown that volatile emission is regulated at the level of gene expression [8], [9], [10], [11]. The well-documented transcriptional regulation of volatile biosynthetic genes during flower development enables us to probe the extent of co-expression and transcriptional regulation of metabolic pathways that provide precursors for volatile biosynthesis.

In this study we show that substantial re-arrangements in gene expression and metabolite abundance take place during flower development. Expression of genes involved in photosynthesis, cell wall formation and fatty acid biosynthesis was down-regulated upon flower opening, whereas the shikimate/phenylpropanoid and isoprenoid pathways were up-regulated. There was a stark contrast in developmental gene expression profiles between volatile biosynthetic pathways and glycolysis and the pentose phosphate pathway, both of which provide precursors for volatiles. Lastly, we demonstrate that transcriptional up-regulation of the volatile biosynthetic pathways is not limited to their last biochemical step, but rather extends to the first committed step in these pathways.

## Results

### Petals Undergo Major Phenotypic and Metabolic Changes During Flower Development

**Figure 1:**
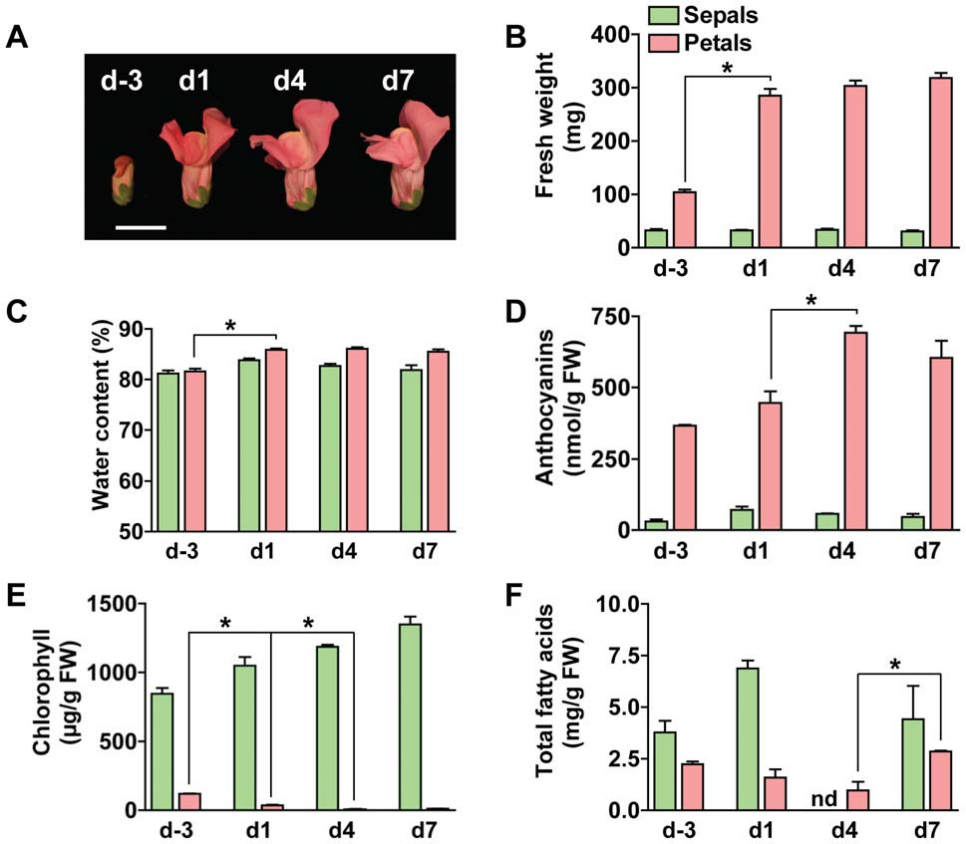
Changes in phenotype and biomass composition during snapdragon flower development. (**A**) Image of developmental stages used for this study. Scale bar = 2 cm. (**B**) Fresh weight, (**C**) water content, (**D**) anthocyanin, (**E**) chlorophyll, and (**F**) total fatty acid content in petals and sepals at different stages of development. Data are means ± SE (*n* = 2−5 biological replicates). * indicates significant changes (p-value<0.05) as compared to the preceding developmental stage within the given tissue.

To investigate developmental changes in the flower metabolic network, snapdragon flower development was divided into four successive stages, each three days long, representative of distinct developmental events: (i) preanthesis, (ii) anthesis, (iii) maturation, and (iv) presenescence. Preanthesis included flower buds from four to two days before flower opening; anthesis consisted of flowers from one day before anthesis to two days postanthesis; maturation comprised three to five day-old flowers; and presenescence consisted of six to eight day-old flowers. Petal and sepal samples were harvested at the middle of each stage: on day three before opening for the preanthesis stage (d-3); on the day of anthesis (d1) for the anthesis stage; on day four postanthesis for the maturation stage (d4) and on day seven postanthesis for the presenescence stage (d7) (Figure 1A). To examine developmental events occurring during the four stages described above, a series of phenotypic traits were measured.

While sepal fresh weight did not change over flower development, petal fresh weight significantly increased from d-3 to d1 and remained unchanged thereafter (Figure 1B). The increase in petal fresh weight was mainly due to an increase in biomass, since the total water content was increased by only 5% after flower opening (Figure 1C). In petals, anthocyanin content gradually increased, reaching the highest level on d4, while chlorophyll levels declined by more than 91% percent from d-3 to d4 and d7 (Figure 1D and 1E). In contrast to petals, chlorophyll levels in sepals gradually increased until d7, whereas the anthocyanin content was low and steady over flower development (Figure 1D and E). As illustrated in Figure 1F, total fatty acid levels (including both free and esterified fatty acids) slightly decreased in petals between d-3 and d4 and increased during presenescence (d7). As compared to petals, total fatty acid levels in sepals did not significantly change over development (Figure 1F).

### Transcriptome Analysis Reveals Major Differences between Developmental Gene Expression Profiles of Petals and Sepals

**Figure 2:**
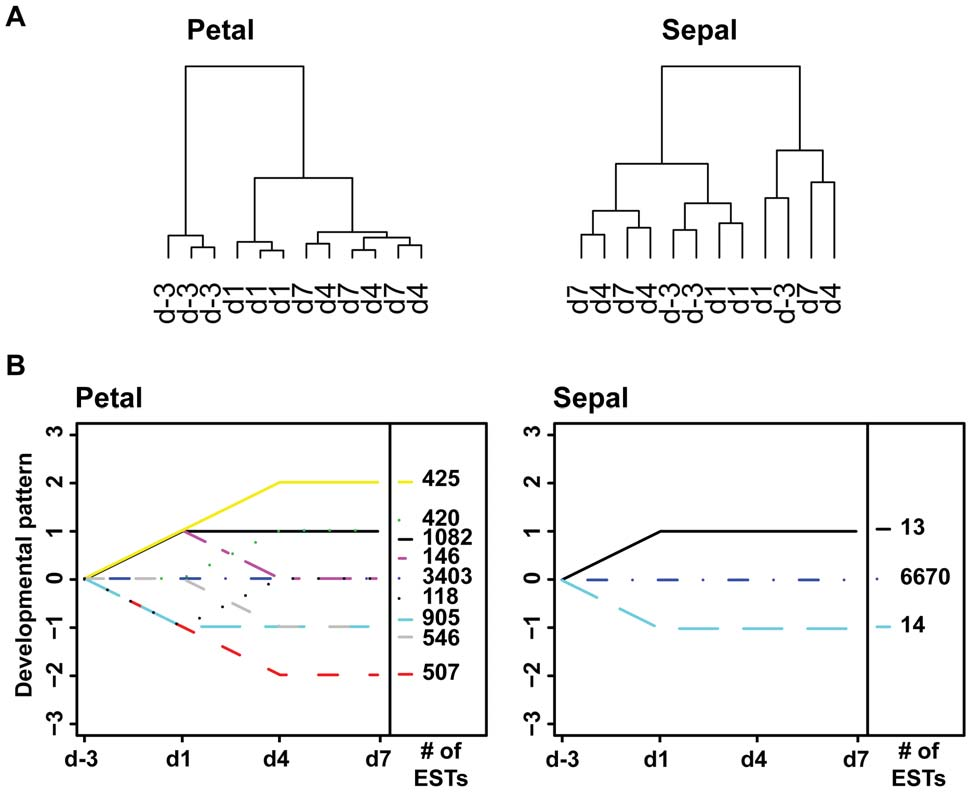
Developmental rearrangements in gene expression of snapdragon petals and sepals. (**A**) Cluster dendrogram showing samples collected at the four stages of petal and sepal development. Samples were clustered according to their gene expression profile. (**B**) Grouping of expressed sequence tags (ESTs) based on their developmental profile. Using a one-way ANOVA, three hypotheses were tested: H_0_: µ_d-3_ = µ_d1_, H_0_: µ_d1_ = µ_d4_, H_0_: µ_d4_ = µ_d7_. A score was attributed for each test. If the gene expression was not significantly different (p-value>0.05), the score = 0. If it was significantly up-regulated (p-value<0.05), the score = 1. If it was significantly down-regulated (p-value<0.05), the score = −1. Genes were grouped based on the combination of scores for the three tests. The number of ESTs in each developmental pattern is indicated next to the graphs. Developmental expression profiles containing less than ten ESTs are not represented.

To analyze transcriptomic changes occurring within non-photosynthetic petals and photosynthetic sepals over flower development, RNA was isolated from petal and sepal tissues at each developmental stage (three biological replicates, 24 samples total) and hybridized to custom-made oligonucleotide microarrays containing probe sets representing 11,959 unigenes found in an *Antirrhinum majus* expressed sequence tag (EST) database [18]. Microarray data were normalized using the robust multiarray average approach [19]. Interquartile range filtering was applied in order to eliminate probe sets with low variation across arrays. Filtering removed 4384 probe sets in petals and 5238 in sepals. Filtered data were subjected to cluster analysis which revealed that d-3 and d1 petals had distinct gene expression profiles, whereas d4 and d7 formed a separate cluster and could not be distinguished based on gene expression (Figure 2A). Gene expression profiles from adjacent development stages were more similar than between distant stages. In sepals, none of the four stages formed a distinct cluster (Figure 2A), suggesting that overall gene expression profiles did not change over development.

To assess the developmental expression pattern of each EST, a one-way ANOVA with p-value adjustment by the Benjamini-Hochberg procedure [20] was conducted. Three comparisons were made: d-3 *vs.* d1, d1 *vs.* d4, and d4 *vs.* d7 and a three-digit developmental pattern score derived from the results of these comparisons was assigned to each EST. Absence of significant change was set as “0”, whereas “1” and “−1” were attributed for up-regulation and down-regulation, respectively. For example, the developmental pattern score {1,0,0} indicates that gene expression was up-regulated from d-3 to d1 and remained unchanged thereafter.

Grouping of ESTs based on their developmental pattern scores revealed that the expression of the majority of ESTs in petals and sepals did not significantly change over flower development (Figure 2B; 3403 and 6670 ESTs with developmental pattern score {0,0,0} in petals and sepals, respectively). The expression of genes encoding enzymes involved in the final steps of volatile formation in petals (i.e. myrcene synthase, ocimene synthase, nerolidol/linalool synthase, and benzoic acid carboxyl methyltransferase) fell either into the cluster with the developmental pattern score {1,0,0} or into the cluster {1,1,0}. These results are in line with previous Northern blot analysis and quantitative real-time PCR which showed that expression of these genes in petals increases after anthesis and peaks when the flower is ready for pollination (four to seven days after anthesis) [8], [9], [10], [11]. Interestingly, 1507 ESTs displayed the same developmental patterns as genes involved in the biosynthesis of volatiles (Figure 2B), suggesting that almost 13% of all ESTs present on the microarray are co-expressed with scent-related genes. An additional 420 ESTs were up-regulated between d1 and d4 (with the scores {0,1,0}) and contained several genes encoding enzymes responsible for the biosynthesis of precursors for snapdragon volatiles, including the large subunit of geranyl diphosphate synthase (terpenoid biosynthesis), 3-Deoxy-D-*arabino*-heptulosonate 7-phosphate synthase, chorismate synthase, and prephenate aminotransferase (phenylpropanoid biosynthesis). Thus, the total percentage of genes up-regulated during flower development constituted 16% (1927 ESTs) of analyzed ESTs. A similar number of genes (1958 ESTs) were down-regulated, exhibiting the pattern scores {−1,0,0}, {0, −1,0} and {−1, −1,0}. Out of these down-regulated genes, 905 ESTs displayed the pattern score {−1,0,0}, which is inversely proportional with biomass levels. Lastly, 2% of the analyzed ESTs (264 ESTs) were differentially expressed over development and displayed a combination of developmental up- and down-regulation (pattern scores {1, −1,0} and {−1,1,0}). In contrast to petals, very few changes were observed in sepal expression profiles. Only 13 ESTs were up-regulated during anthesis ({1,0,0}) and 14 EST were down-regulated ({−1,0,0}).

To determine biological processes statistically enriched in the developmental clusters that were up-regulated or down-regulated over petal development, a Fisher’s exact test with control of false discovery rate was conducted [21]. This analysis revealed that petal clusters which contain scent related genes and have the developmental pattern scores {1,0,0}, {0,1,0}, and {1,1,0} were enriched in genes that encode enzymes involved in secondary metabolism (i.e., phenylpropanoid and isoprenoid biosynthesis), as well as fatty acid catabolism, amino acid transport, and regulation of cellular biosynthetic processes (Table 1). Clusters containing genes which expression was down-regulated over petal development (with scores {−1,0,0}, {0, −1,0}, and {−1, −1,0}) were enriched in genes that are required for fatty acid biosynthesis, cell wall organization and biogenesis, cell morphogenesis, small molecule metabolic processes and photosynthesis (Table 2).

**Table 1:** Gene Ontology term enrichment analysis for up-regulated genes contained in the petal developmental clusters {0,1,0}, {1,0,0}, and {1,1,0}.

GO term	p-value^a^
Fatty acid β-oxidation	1.48×10^−03^
Response to chitin	1.02×10^−02^
Regulation of cell communication	1.94×10^−02^
Isoprenoid metabolic process	2.19×10^−02^
Regulation of response to stimulus	2.24×10^−02^
Response to salicylic acid stimulus	2.77×10^−02^
Positive regulation of cellular process	3.65×10^−02^
Phenylpropanoid biosynthetic process	3.83×10^−02^
Aromatic amino acid family biosynthetic process	3.83×10^−02^
Response to water	3.83×10^−02^
Photomorphogenesis	4.64×10^−02^
Response to stress	4.65×10^−02^
Neutral amino acid transport	4.71×10^−02^

a GO terms with FDR corrected p-value less than 0.05 are shown.

**Table 2:** Gene Ontology term enrichment analysis for down-regulated genes contained in the petal developmental clusters {0,−1,0}, {−1,0,0}, and {−1,−1,0}.

GO term	p-value^a^
Cellular response to gravity	3.07×10^−07^
Unidimensional cell growth	4.42×10^−07^
Transmembrane receptor protein tyrosine kinase signaling pathway	9.20×10^−07^
Microtubule-based movement	5.87×10^−06^
Plant-type cell wall organization	1.75×10^−05^
Secondary cell wall biogenesis	4.10×10^−04^
Cellulose biosynthetic process	1.90×10^−03^
Hexose metabolic process	2.33×10^−03^
Nucleotide-sugar metabolic process	2.65×10^−03^
Primary cell wall biogenesis	3.10×10^−03^
Syncytium formation	7.39×10^−03^
Cytoskeleton organization	9.47×10^−03^
Photosynthesis, light harvesting in photosystem I	1.04×10^−02^
Cellular carbohydrate catabolic process	1.15×10^−02^
Stomatal complex development	1.62×10^−02^
Protein polymerization	1.95×10^−02^
Cell wall polysaccharide metabolic process	2.22×10^−02^
Pectin metabolic process	2.45×10^−02^
Cellular cell wall macromolecule metabolic process	2.99×10^−02^
Cell wall thickening	3.03×10^−02^
Cutin biosynthetic process	3.42×10^−02^
Trichoblast differentiation	4.36×10^−02^
Steroid biosynthetic process	4.49×10^−02^

a GO terms with FDR corrected p-value less than 0.05 are shown.

### Primary and Secondary Metabolic Pathways Display different Gene Expression Profiles During Petal Development

**Figure 3:**
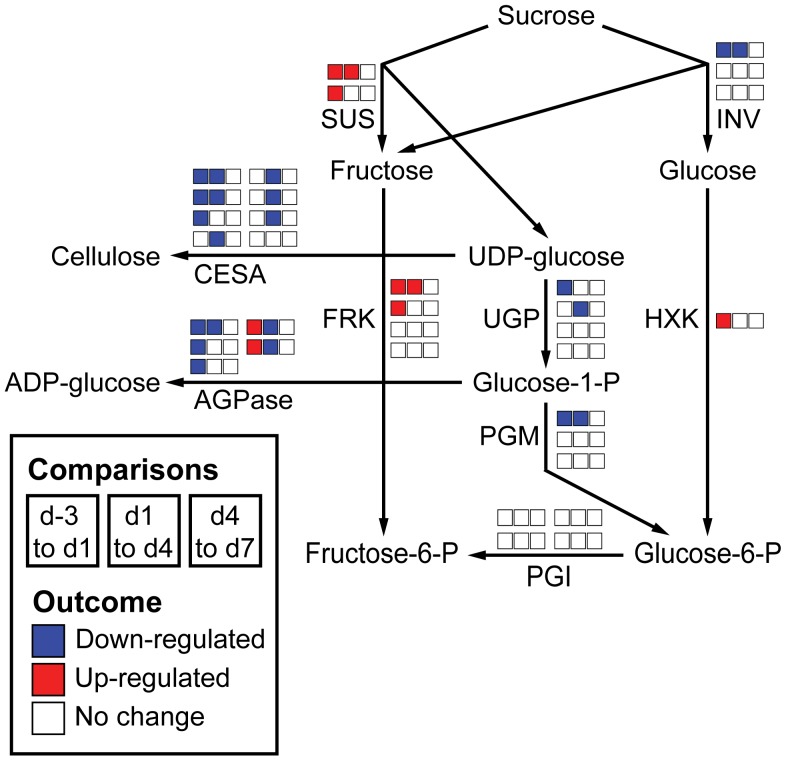
Gene expression in sucrose metabolism over petal development. ESTs were annotated and assigned to enzymatic steps based on their homology to *A. thaliana* genes involved in each enzymatic step. Changes in expression of each EST are represented by three boxes corresponding to the three comparisons: d-3 *vs*. d1, d1 *vs*. d4, d4 *vs*. d7. Depending on the number of identified ESTs, each enzymatic step has a different number of sets of three boxes. The boxes were colored according to the change in gene expression: Red and blue boxes indicate significant up- and down-regulation (p-value<0.05), respectively, for a given comparison, while white boxes indicate no significant change in gene expression (p-value>0.05). AGPase: ADP-glucose pyrophosphorylase, FRK: fructokinase, CESA: cellulose synthase, HXK: hexokinase, INV: invertase, PGI: phosphoglucose isomerase, PGM: phosphoglucomutase, SUS: sucrose synthase, UGP: UDP-glucose pyrophosphorylase.

To examine developmental changes in specific metabolic pathways, ESTs and their respective expression profiles (based on the outcome of the ANOVA described above) were mapped onto different metabolic pathways (Figures 3–5). Annotation of snapdragon ESTs was performed based on the amino acid sequence homology (tblastx) to *Arabidopsis thaliana* proteins involved in the examined pathways.

**Figure 4 pone-0040381-g004:**
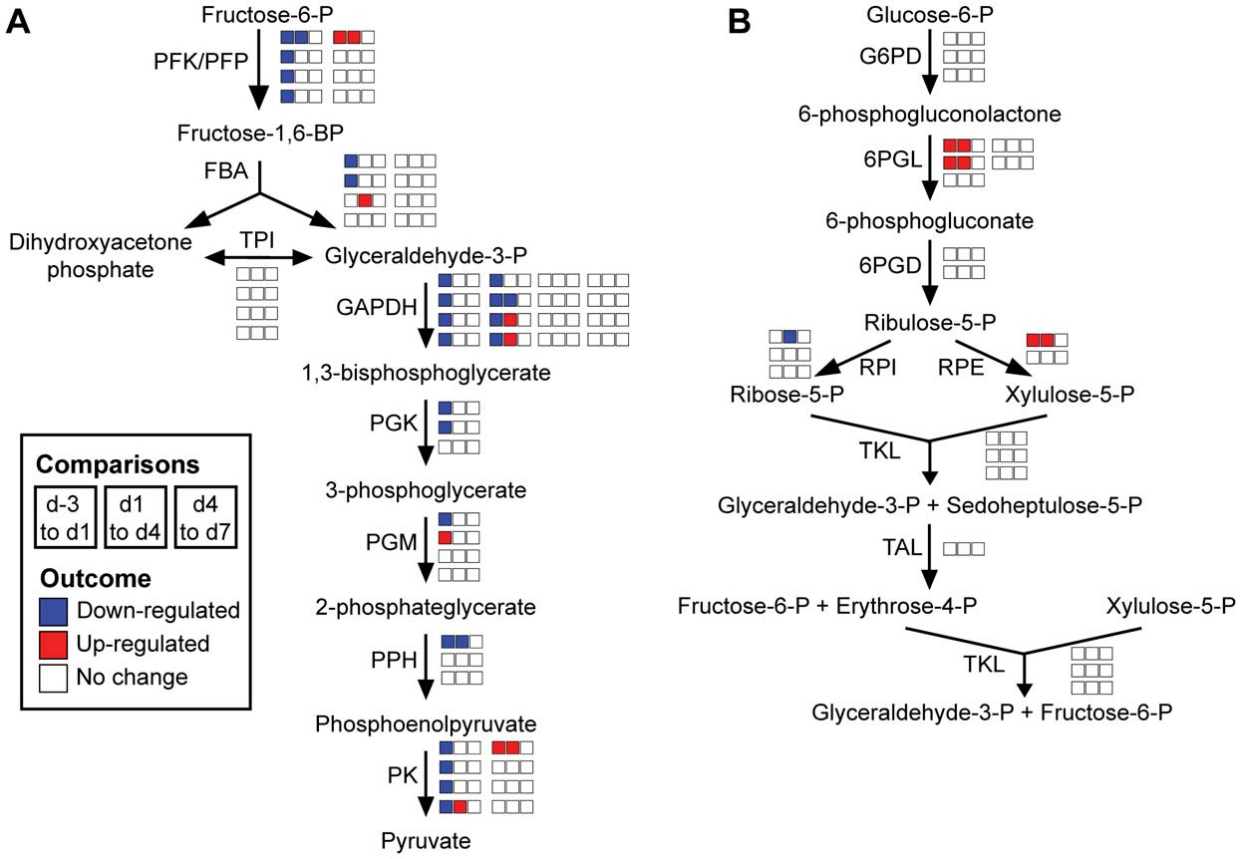
Gene expression in glycolysis and the pentose phosphate pathway over petal development. Developmental gene expression changes in (**A**) glycoylsis and the (**B**) pentose phosphate pathway are illustrated as in Figure 3. 6PGD: 6-phosphogluconate dehydrogenase, 6PGL: 6-phosphogluconolactonase, FBA: fructose-bisphosphate aldolase, G6PD: glucose-6-phosphate dehydrogenase, GAPDH: glyceraldehyde-3-phosphate dehydrogenase, PFK/PFP: phosphofructokinase/6-phosphofructo-1-phosphotransferase, PGK: phosphoglycerate kinase, PGM: phosphoglycerate mutase, PK: pyruvate kinase, PPH: phosphopyruvate hydratase, RPE: ribulose-phosphate 3-epimerase, RPI: ribose-5-phosphate isomerase, TAL: transaldolase, TKL: transketolase, TPI: triose-phosphate isomerase.

**Figure 5:**
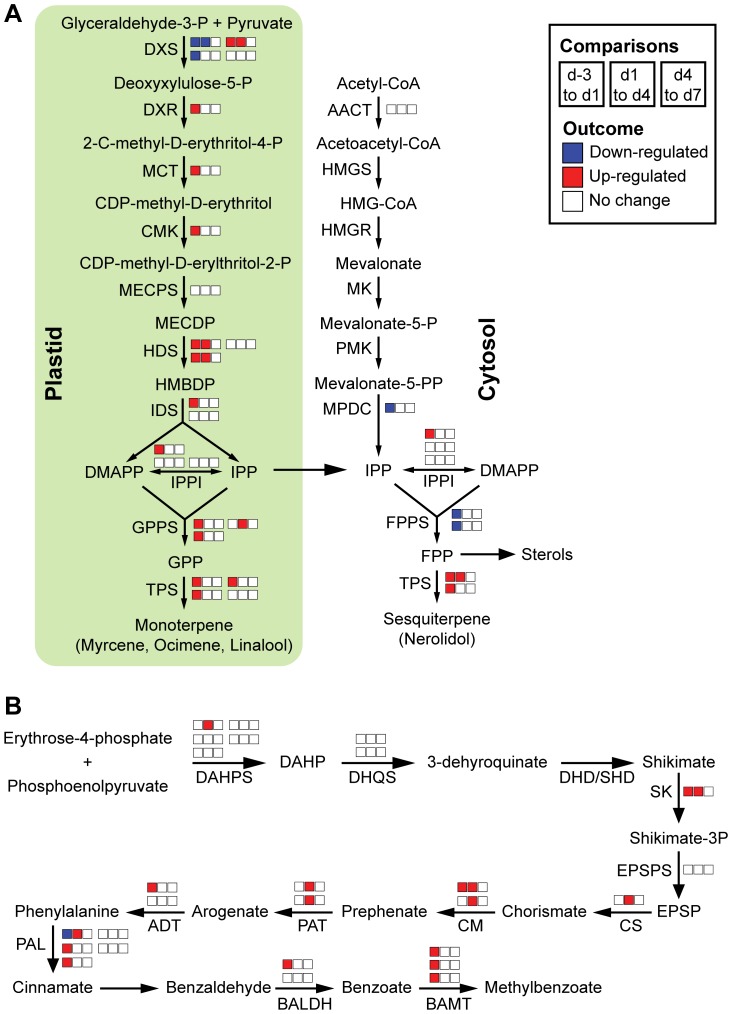
Developmental gene expression changes in pathways leading to petal volatile formation. Developmental gene expression changes in the (**A**) methylerythritol phosphate (MEP) pathway, the (**B**) mevalonic acid (MVA) pathway, and the (**C**) shikimate/phenylpropanoid pathway are illustrated as in Figure 3. AACT: acetyl-CoA acetyltransferase, ADT: arogenate dehydratase, BALDH: benzaldehyde dehydrogenase, BAMT: S-adenosyl-L-methionine:benzoic acid carboxyl methyltransferase, CM: chorismate mutase, CMK: 4-(cytidine 5′-diphospho)-2-C-methyl-D-erythritol kinase, CS: chorismate synthase, DAHP: 3-deoxy-arabino-heptulonate 7-phosphate, DAHPS: 3-deoxy-7-phosphoheptulonate synthase, DHD/SHD: dehydroquinate dehydratase/shikimate dehydrogenase, DHQS: 3-dehydroquinate synthase, DMAPP: dimethylallyl pyrophosphate, DXS: 1-deoxy-D-xylulose 5-phosphate, DXR: 1-deoxy-D-xylulose-5-phosphate reductoisomerase, EPSP: 3-enolpyruvyl-shikimate-5-phosphate, EPSPS: 3-phosphoshikimate 1-carboxyvinyltransferase, FPP: farnesyl pyrophosphate, FPPS: farnesyl pyrophosphate synthase, GPP: geranyl pyrophosphate, GPPS: geranyl pyrophosphate synthase, HDS: 4-hydroxy-3-methylbut-2-en-1-yl diphosphate synthase, HMBDP: (E)-4-hydroxy-3-methylbut-2-en-1-yl diphosphate, HMG-CoA: hydroxymethylglutaryl-CoA, HMGR: hydroxymethylglutaryl-CoA reductase, HMGS: hydroxymethylglutaryl-CoA synthase, IDS: isopentenyl diphosphate synthase, IPP: isopentenyl pyrophosphate, IPPI: isopentenyl pyrophosphate isomerase, MCT: 2-C-methyl-D-erythritol 4-phosphate cytidylyltransferase, MECPD: 2-C-methyl-D-erythritol 2,4-cyclodiphosphate, MECPS: 2-C- methyl-D-erythritol 2,4-cyclodiphosphate synthase, MK: mevalonate kinase, MPDC: mevalonate diphosphate decarboxylase, PAL: phenylalanine ammonia lyase, PAT: prephenate aminotransferase, PMK: phosphomevalonate kinase, SK: shikimate kinase, TPS: terpene synthase.

#### Sucrose metabolism

The starting point for flower metabolism is sucrose, the primary carbon source for primary and secondary metabolites, which is degraded by sucrose synthase and/or invertase to generate precursors for the oxidative pentose phosphate pathway and glycolysis (Figure 3). Analysis of sucrose synthase and invertase expression in snapdragon petals showed that they exhibited opposite developmental trends (Figure 3). While sucrose synthase expression was up-regulated from d-3 to d4, expression of invertase decreased. ESTs encoding UDP-glucose pyrophosphorylase and phosphoglucomutase, which are required for the biosynthesis of glucose-6-phosphate, were down-regulated. Since expression of ESTs corresponding to fructokinase resembled that of sucrose synthase and was upregulated, our results suggest that the formation of fructose-6-phospate via the sucrose synthase/fructokinase route becomes more prominent over petal development in snapdragon flowers.

Cellulose synthase (CESA) and ADP-glucose pyrophosphorylase (AGPase) use UDP-glucose and glucose-1-phosphate as respective substrates and direct carbon flux toward the formation of cell wall and starch, respectively. Consistent with slower growth of petals after anthesis (Figure 1A and 1B), expression of ESTs encoding CESA and AGPase decreased during petal development (Figure 3).

#### Glycolysis and the pentose phosphate pathway

Glycolysis and the pentose phosphate pathway generate energy and reducing equivalents and provide precursors to various pathways leading to primary and secondary metabolites. The major secondary metabolites produced in snapdragon flowers (i.e., anthocyanin pigments and volatiles) are derived from the phenylpropanoid and terpenoid pathways, both of which rely on glycolysis and pentose phosphate pathway for supply of precursors.

The majority of differentially expressed ESTs involved in glycolysis exhibited a decrease in expression from d-3 to d1 (Figure 4A). In contrast to glycolysis, little changes were observed in the expression of snapdragon ESTs involved in the pentose phosphate pathway (Figure 4B). Only expression of 6-phosphogluconate dehydrogenase (6PGL) and ribulose 5-phosphate 3-epimerase (RPE) increased from d-3 to d4 (Figure 4B).

#### Pathways involved in volatile and anthocyanin production

Volatiles emitted from snapdragon flowers are derived from both the shikimate/phenylpropanoid and the mevalonic acid (MVA)/methylerythritol phosphate (MEP) pathways. The emission of these volatiles and the expression of genes responsible for the final steps of their biosynthesis are developmentally orchestrated [8], [9], [10], [11]. Previous studies have also shown that the levels of substrates [9], their subcellular availability [11], and the activity of scent biosynthetic enzymes [9] contribute to the regulation of scent production. The microarray analysis performed here allowed us to evaluate the expression of genes involved not only in the final steps of scent formation, but also in the entire pathways leading to the synthesis of floral scent precursors.

Prior studies showed that the MEP pathway provides isopentenyl diphosphate (IPP) precursors for both plastidial monoterpene and cytosolic sesquiterpene biosynthesis in the epidermis of snapdragon petals [22]. Expression analysis of genes involved in the MEP pathway revealed that at least one EST per biochemical reaction was up-regulated over flower development (Figure 5A), with the exception of 2-C-methyl-D-erythritol 2,4-cyclodiphosphate synthase (MECPS), the expression of which remained unchanged (Figure 5A). The first committed step in the MEP pathway, the condensation of pyruvate and glyceraldehyde 3-phosphate, is catalyzed by 1-deoxy-D-xylose-5-phosphate synthase (DXS). Out of four DXS ESTs, only one was up-regulated. This EST was previously reported to display tissue-specific and rhythmic expression profiles typical for “scent” genes and to modulate the carbon flux through the MEP pathway [22]. Consistent with the minor contribution of the MVA pathway to sesquiterpene volatile biosynthesis in snapdragon flowers [22], only two ESTs encoding enzymes of the MVA pathway were present in the 11,959 probe sets and their expression was unchanged or down-regulated over petal development (Figure 5A). As the MEP pathway generates IPP and DMAPP at a 6∶1 ratio [23], [24] and the MVA pathway produces only IPP, IPP isomerase (IPPI) activity is required to optimize the relative amounts of IPP and DMAPP for efficient terpenoid biosynthesis. Our results show that one IPPI was up-regulated in petals from d-3 to d1.

The next step in monoterpene and sesquiterpene biosynthesis involves the formation of prenyl diphosphate precursors, geranyl diphosphate (GPP) and farnesyl diphosphate (FPP), respectively, by the head-to-tail condensation of IPP(s) and DMAPP in reactions catalyzed by corresponding short-chain prenyltransferases, such as GPP synthase (GPPS) and FPP synthase (FPPS). While down-regulation was observed for the FPPS ESTs (Figure 5A), GPPS ESTs were up-regulated over petal development. It should be noted that snapdragon flowers possess a heterodimeric GPPS consisting of a catalytic large subunit (GPPS.LSU) and an inactive small subunit (GPPS.SSU) that controls the rate of GPP production, and thus monoterpene biosynthesis [25]. Expression of the two ESTs corresponding to GPPS.SSU was up-regulated from d-3 to d1 and remained unchanged thereafter, whereas the GPPS.LSU EST exhibited a {0,1,0} developmental pattern. These expression patterns are similar to developmental expression profiles previously determined by Northern blot [25]. The expression of terpene synthases, which catalyze the conversion of GPP and FPP to volatile terpenoids was up-regulated upon anthesis. These expression profiles were similar, but not identical to previously described developmental expression of snapdragon terpene synthases, likely due to some limitations of microarray analysis, which is known to have a decreased ability to resolve changes in the expression of highly abundant genes. Moreover, due to high homology of terpene synthase ESTs to previously identified myrcence synthase, ocimene synthase [8], and linalool/nerolidol synthase [11] genes, it was impossible to clearly assign ESTs to a particular gene.

The shikimate and phenylalanine biosynthetic pathways link primary metabolism and phenylpropanoid volatile/anthocyanin biosynthesis. The expression of most of the ESTs involved in the shikimate/phenylalanine pathway and phenylpropanoid volatile biosynthesis displayed orchestrated up-regulation from d-3 to d1 or d4 (Figure 5B). Similar to expression of terpene synthase ESTs, expression of ESTs corresponding to benzaldehyde dehydrogenase and benzoic acid carboxyl methyltransferase, which are responsible for the biosynthesis of benzoic acid and methylbenzoate respectively, was similar but not identical to their previously characterized developmental expression profiles [9], [10]. Several ESTs in anthocyanin production were down-regulated during snapdragon flower development, consistent with previous data showing that anthocyanin formation begins prior to flower opening ([5]; Figure S1). Only one EST corresponding to naringenin chalcone synthase (CHS) was up-regulated from d-3 to d4 (Figure S1).

### Petals Display High Chemodiversity and Undergo Extensive Metabolic Remodeling During Flower Development

**Figure 6:**
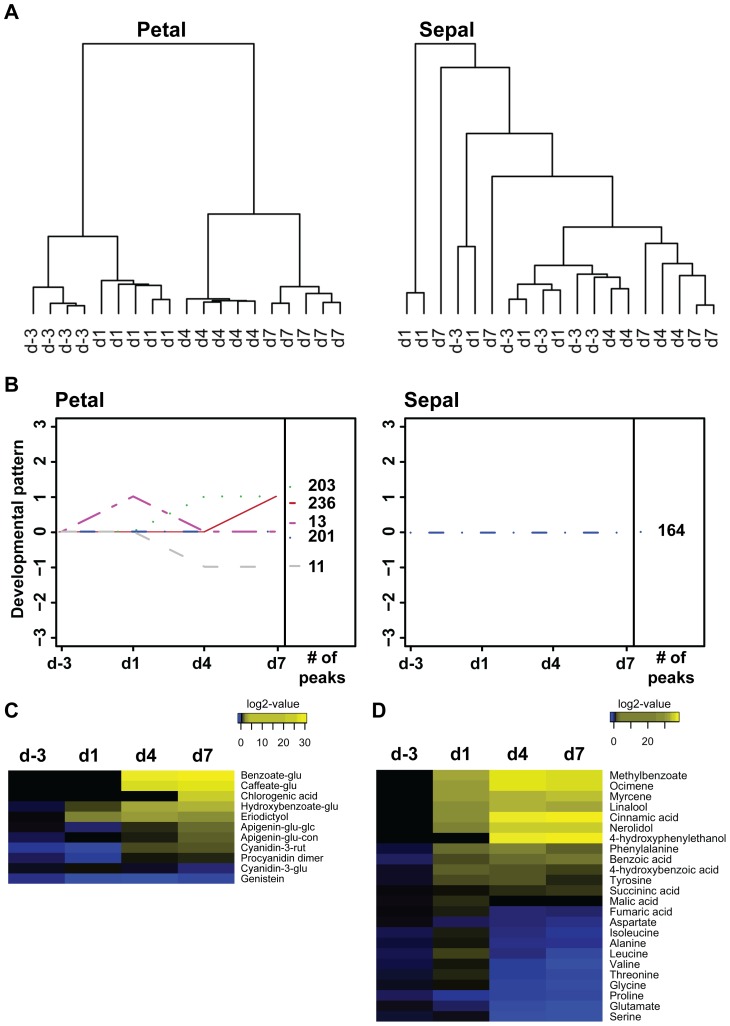
Remodeling of the petal and sepal metabolomes over flower development. (**A**) Cluster dendrogram showing the four stages of petal and sepal development. Samples were clustered according to their individual metabolomic profiles as measured by non-targeted liquid chromatography-mass spectrometry (LC-MS). (**B**) Grouping of peaks detected by non-targeted LC-MS analysis based on their developmental profile. Using a one-way ANOVA, three hypotheses were tested: H_0_: µ_d-3_ = µ_d1_, H_0_: µ_d1_ = µ_d4_, H_0_: µ_d4_ = µ_d7_. A score was attributed for each test. If the gene expression was not significantly different (p-value>0.05), the score = 0. If it was significantly up-regulated (p-value<0.05), the score = 1. If it was significantly down-regulated (p-value<0.05), the score = −1. Genes were grouped based on the combination of scores for the three tests. The number of peaks in each developmental pattern is indicated next to the graphs. Heatmap of (**C**) metabolites putatively identified by ion trap LC-MS^n^ and of (**D**) metabolites measured by gas chromatography-mass spectrometry in snapdragon petals. Metabolite levels were expressed relative to the average value for that metabolite in d-3 samples and log_2_-transformed. Log_2_-transformed values were averaged for each stage. Yellow indicates an increase in metabolite abundance and blue a decrease.

To follow metabolic changes occurring over flower development in petals and sepals, we performed extensive metabolic profiling using non-targeted liquid chromatography-mass spectrometry (LC-MS) and targeted gas chromatography-mass spectrometry (GC-MS). Metabolites were isolated from petals and sepals at each developmental stage in four to five biological replicates (38 samples total) and subjected to LC-MS analysis. Baseline subtraction, alignment, peak-picking, and normalization were applied for the pre-processing of LC-MS data (GeneSpring MS, Agilent Technologies). More metabolites were detected in petals (664 peaks) than in sepals (164 peaks). Hierarchical clustering of petal metabolite profiles revealed clear differences between the developmental stages, with adjacent stages being more similar to each other than distant ones (Figure 6A). In contrast, there was no clear separation between metabolite profiles in sepals at different developmental stages (Figure 6A), suggesting that developmental reconfiguration of the metabolome occurs exclusively in petals. Grouping of metabolites according to their developmental pattern score, using the same statistical approach as for transcriptome analysis, revealed no changes in metabolite abundance in sepals over development (Figure 6B). In contrast, 70% of petal metabolites significantly changed over development with 439 metabolites increasing during flower development, ({0,1,0} or {0,0,1} pattern score; Figure 6B).

Analysis of volatile emission over flower development revealed that emitted compounds exhibit a {0,1,0} pattern [8], [9], [10], [11]. A similar pattern was observed for anthocyanin levels over petal development based on the data presented in Figure 1D. To determine the chemical identity of metabolites which show developmental changes similar to those observed for scent compounds and anthocyanins (with a {0,1,0} developmental pattern score), several peaks were tentatively identified based on their retention time, mass-to-charge ratio (m/z), and fragmentation pattern using ion-trap LC-MS^n^ (Figure 6C). Identified compounds appear to derive from Phe and include a range of glycosylated benzoic and hydroxycinnamic acid derivatives, free (eriodictyol, procyanidin, and genistein) and conjugated (apigenin-coniferaldehyde glucoside, apigenin-glucoside-glucuronide) flavonoids, and glycosylated cyanidins (cyanidin-3-rutinoside and cyanidin-3-glucoside) (Figure 6C).

Targeted metabolic profiling of petal tissue by GC-MS revealed that the detected primary and secondary metabolites fell into different classes based on their developmental abundance profiles (Figure 6D). One class that consists of terpenoid volatiles, tyrosine, phenylalanine, and their derivatives was characterized by expansion of metabolite pools from d-3 to d4 and near-constant pools from d4 to d7. Another class included TCA cycle intermediates. The pools of succinate constantly increased from d-3 to d7, whereas fumarate and malate levels increased from d-3 to d1 and dropped to levels comparable to d-3 thereafter. While the aromatic amino acids phenylalanine and tyrosine increased over petal development, other detected free amino acids showed more complex changes and formed a third class. Their abundance increased from d-3 to d1 and decreased thereafter, for some amino acids even below the levels at d-3 (Figure 6D).

### Integrative Analysis of Transcriptomic and Metabolomic Data Uncovers Functional Relationships between Transcripts and Metabolites

**Figure 7:**
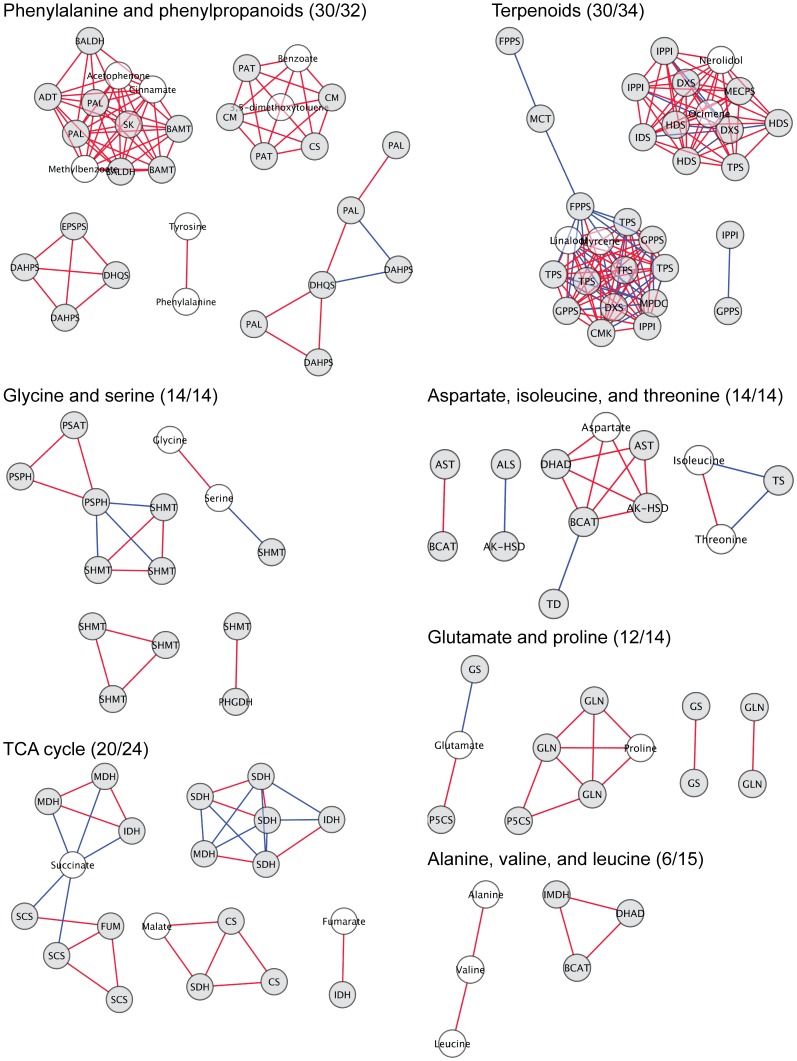
Gene to metabolite correlation network for amino acid pathways, the TCA cycle, and the phenylporpanoid and terpenoid network. Gene-to-metabolite networks in snapdragon petals. ESTs are represented by gray circles and metabolites by white circles. Red lines indicate positive correlations (*r*>0.95, p-value<0.05) and blue lines negative correlations (r<0.95, p-value<0.05). Numbers in parentheses describe the numbers of nodes (metabolites or ESTs) being correlated to one or more nodes and the total number of nodes in the pathway.

To obtain comprehensive insights into co-regulatory relationships between transcripts and metabolites, we performed an integration of the petal transcriptome and metabolome data sets. Correlation between genes and metabolites in amino acid pathways, TCA cycle, and the terpenoid pathways was assessed using the Cytoscape plugin Expression Correlation Network [26]. The results showed that in most cases changes in metabolite levels and expression of genes in the biosynthetic pathway leading to the corresponding metabolites were positively correlated (Figure 7). Interestingly, genes and metabolites in the shikimate/phenylalanine and phenylpropanoid pathways, as well as in the terpenoid pathways were highly correlated with each other. TCA cycle genes and metabolites also exhibited a high degree of correlation between their developmental expression and abundance profiles. Genes and metabolites in other amino acid pathways were correlated to a lesser extent (Figure 7).

To analyze whether genes and metabolites that exhibit the same developmental profile are involved in related biological processes, we performed a gene ontology (GO) term enrichment analysis with the genes positively correlated to metabolites. This analysis revealed that genes correlated with metabolites in the “Phenylalanine, tyrosine, and phenylpropanoids” and “Terpenoids” classes were enriched for similar biological processes (see Table S1 for metabolite class assignment of metabolites). Indeed, both classes contained GO terms related to transcriptional regulation, protein degradation, autophagy, fatty acid β-oxidation, and remobilization of nutrients (Table 3). TCA cycle intermediates were linked to flavonol biosynthesis, cellular catabolism, protein ubiquitination, and response mechanisms to extracellular stimulus. Amino acids other than phenylalanine and tyrosine were associated with cell wall biosynthesis, cuticle formation, and cell growth.

**Table 3:** Gene ontology terms enriched in genes that show significant positive correlation with the metabolite classes “Phe, Tyr, phenylpropanoids”, “Terpenoids”, “TCA cycle intermediates”, and “AA”.

Metabolite class	GO term	p-value^a^
Phenylalanine, tyrosine, and phenylpropanoids	Ubiquitin-dependent protein catabolic process	5.48×10^−06^
	Protein ubiquitination	1.07×10^−04^
	Fatty acid β-oxidation	1.35×10^−03^
	Response to chitin	1.64×10^−03^
	Autophagy	3.76×10^−03^
	Iron-sulfur cluster assembly	8.60×10^−03^
	Peroxisome organization	9.47×10^−03^
	Dipeptide transport	1.67×10^−02^
	Positive regulation of transcription, DNA-dependent	1.90×10^−02^
	Cellular response to stimulus	2.13×10^−02^
	Phosphorus metabolic process	3.02×10^−02^
	Regulation of cell communication	3.54×10^−02^
	Tripeptide transport	3.76×10^−02^
	Post-translational protein modification	3.76×10^−02^
	Negative regulation of response to stimulus	4.04×10^−02^
	Inorganic anion transport	4.24×10^−02^
Terpenoids	Regulation of transcription, DNA-dependent	9.03×10^−04^
	Iron-sulfur cluster assembly	1.32×10^−03^
	Fatty acid β-oxidation	2.11×10^−03^
	Cellular response to stimulus	8.05×10^−03^
	Response to chitin	1.32×10^−02^
	Negative regulation of response to stimulus	2.32×10^−02^
	Peroxisome organization	3.15×10^−02^
	Positive regulation of macromolecule metabolic process	3.28×10^−02^
	Histone H2B ubiquitination	3.33×10^−02^
	Autophagy	3.99×10^−02^
	Ubiquitin-dependent protein catabolic process	4.36×10^−02^
	Phosphorus metabolic process	4.59×10^−02^
	Regulation of cell communication	4.77×10^−02^
TCA cycle intermediates	Flavonol biosynthetic process	1.07×10^−02^
	Cellular catabolic process	3.50×10^−02^
	Cellular response to extracellular stimulus	4.72×10^−02^
	Protein ubiquitination	4.72×10^−02^
Amino acids	Plant-type cell wall modification during multidimensional cell growth	5.64×10^−04^
	Syncytium formation	3.02×10^−03^
	Unidimensional cell growth	3.13×10^−03^
	Wax biosynthetic process	3.34×10^−03^
	Cuticle development	5.60×10^−03^
	alkane biosynthetic process	1.08×10^−02^
	Cellular polysaccharide biosynthetic process	2.68×10^−02^
	Plant-type cell wall loosening	2.92×10^−02^
	Aldehyde catabolic process	3.91×10^−02^

a GO terms with FDR corrected p-value less than 0.05 are shown.

## Discussion

Reproduction and evolutionary success of angiosperms relies on pollinator attraction, which is mediated by visual and olfactory cues [27], [28]. Visual cues usually involve pigment formation, commonly anthocyanin pigments, the levels of which are developmentally regulated [29]. In snapdragon flowers, anthocyanin formation begins before flower opening, where high levels of expression of anthocyanin biosynthetic genes were found [5]. The expression of these genes decreases at flower opening and has not been examined at later stages of flower development. While genes encoding many enzymes in the anthocyanin pathway have been isolated, previous work on floral volatiles has mainly concentrated on the isolation and characterization of enzymes and genes involved in the final steps of scent biosynthesis [5], [8], [9], [10], [11]. As a result, our understanding of the developmental regulation of pathways providing precursors for volatile and anthocyanin biosynthesis remains limited. Here, we provide system-wide insights into transcriptional and metabolic changes occurring during flower development in petals and sepals. Our study highlights the developmental regulation of primary and secondary metabolic pathways and reveals co-regulatory relationships between genes and metabolites within these pathways. Extensive remodeling of transcriptional and metabolite profiles was observed over flower development in petals, but not in sepals, suggesting that petals and sepals are vastly independent in terms of the developmental regulation of metabolism.

### Metabolic Changes Occurring during Flower Development

Analysis of transcriptomic and metabolomic data revealed that over flower development petal metabolism is subjected to important biochemical switches. Petal development in snapdragon flowers is accompanied by an increase in biomass and anthocyanin levels and a decrease in chlorophyll and total fatty acids (Figure 1). While an increase in biomass during transition from the bud stage to open flowers requires the production of macromolecular compounds and energy, the reduction in biomass accumulation after d1 of petal development could be the result of down-regulation of genes and processes involved in organ growth. Analysis of biological processes enriched in the clusters containing ESTs which are down-regulated over petal development support this hypothesis. Indeed, enriched gene ontology terms were related to cell growth, cell wall synthesis, small molecule biosynthesis, fatty acid biosynthesis, and photosynthesis (Table 2). These results were consistent with earlier studies in *A. thaliana*, which show down-regulation of genes involved in cell organization and cell wall precursor biosynthesis in petals between the bud stage and flower opening [30]. The observed developmental changes in fatty acid, anthocyanin, and chlorophyll levels (Figure 1) were not specific only for snapdragon petals. In *Nicotiana tabacum* (tobacco) total fatty acid content decreases from the bud stage to mature flowers and slightly increases in senescing petals [31]. The levels of anthocyanins and chlorophyll in tobacco flowers also display developmental profiles similar to those of snapdragon petals [29]. It has been shown that due to the presence of chlorophyll in buds, tobacco petals are capable of fixing CO_2_ by photosynthesis before anthesis and lose this capacity as chlorophylls are degraded [29]. CO_2_ fixation in the petals of flower buds most likely complements imported sucrose to build resources required for a very rapid flower growth just before anthesis and thus could be a characteristic feature of the early stages of flower development. Down-regulation of photosynthetic processes observed in *A. thaliana* petals [30] as well as in snapdragon petals (Table 2) provides further evidence for a transition from mixotrophy to heterotrophy during flower opening.

After anthesis, snapdragon petals undergo preparation for reproduction, which is accompanied by the generation of visual and olfactory cues for pollinator attraction [8], [9], [10], [11]. Both anthocyanins (Figure 1D) and volatile compounds (Figure 6D; [8], [9], [10], [11]) reach their maximum levels at the maturation stage (d4) when flowers are ready for pollination. Expression analysis of genes leading to biosynthesis of floral volatiles (i.e. the shikimate/phenylalanine, phenylpropanoid, and MEP pathways) revealed their coordinated up-regulation over petal development (Figure 5). Since in snapdragon flowers the plastid-localized MEP pathway provides IPP precursors for both plastidial monoterpene and cytosolic sesquiterpene biosynthesis [22], the observed down-regulation of expression of genes involved in the MVA pathway (Figure 5A) suggests that the main function of the MVA pathway lies in the synthesis of sterols. Enrichment of the gene ontology term “Steroid biosynthetic process” in the clusters containing down-regulated genes (Table 2) further indicates that sterol biosynthesis is down-regulated over petal development. In *Rosa hybrida* petals, it has been shown that sterol levels increase during the early growth phase of petals and remain stable thereafter [32]. As FPP synthase is responsible for the formation of FPP for sesquiterpene, triterpene, and sterol biosyntheses, the observed down-regulation of FPP synthase expression from d-3 to d1 (Figure 5A) indicates that higher levels of FPP synthase protein are required for sterol biosynthesis than for sesquiterpene formation in snapdragon petals.

Surprisingly, GO term enrichment analysis of genes correlating with different metabolite classes revealed the correlation between the “Terpenoids” and “Phenylalanine, tyrosine, and phenylpropanoids” metabolite classes and biological processes involved in senescence [3], such as autophagy, ubiquitin-dependent protein degradation, fatty acid β-oxidation, and nutrient remobilization (Table 3). These results suggest that the onset of senescence occurs long before visible senescence symptoms. In addition, the list of genes correlating with levels of terpenoids and phenylpropanoids over flower development was enriched in genes involved in the regulation of transcription (Table 3). To date, only two the transcription factors ODO1 and EOBII, which control benzenoid volatile emission, have been isolated and characterized from petunia flowers [33], [34]. Thus, the obtained gene list might represent an excellent source for candidates as regulators of scent emission.

### Relationships between Primary and Secondary Metabolic Pathways

Gene ontology term enrichment analysis of clusters containing developmentally up-regulated genes in petals revealed an overrepresentation of genes associated with isoprenoid metabolism, and aromatic amino acid and phenylpropanoid biosyntheses (Table 1). Correlation analysis showed extensive co-expression of genes in volatile biosynthetic pathways - from the first committed step to the last step of the volatile formation (Figure 6A, 6B, and 7). In addition, a positive correlation was found between expression levels of genes involved in these pathways and abundances of pathway-related metabolites (Figure 7). Taken together, these results imply that the formation of volatile biosynthetic pathway intermediates and end-products is mainly regulated at the transcriptional level in snapdragon petals. Similarly, an induction of shikimate and phenylpropanoid pathway gene expression was observed after anthesis in *Brunfelsia* flowers, which produce phenylpropanoid volatiles [35]. In contrast to volatile biosynthetic pathways, genes involved in the anthocyanin formation were unchanged or down-regulated (Figure S1), suggesting that pigment accumulation in mature snapdragon flowers (Figure 1D) is primarily controlled postranscriptionally. However, we do not exclude that yet uncharacterized anthocyanin biosynthetic gene homologs, which were not present on the microarray, might contribute to the late pigment accumulation.

Interestingly, we found an expansion in most flavonoid pools over snapdragon petal development (Figure 6C), as was also observed in *Brunfelsia* flowers [35]. Flavonoids assume various roles in plant tissues, notably as mediators of plant-pollinator interactions, antioxidants, and protectants against ultraviolet radiation [36]. The similarity between flavonoid developmental profiles and anthocyanins/volatile compounds points to the major function of flavonoids in pollinator attraction in snapdragon petals.

Secondary metabolites produced in plant cells are ultimately derived from products of primary metabolism. In snapdragon, glycolysis and the pentose phosphate pathway provide precursors for volatile terpenoid and phenylpropanoid formation, as well as anthocyanin production. To determine whether transcriptional changes in glycolysis and the pentose phosphate pathway reflect increased demand for secondary metabolite precursors over flower development, we analyzed the developmental gene expression in these pathways. The majority of ESTs in glycolysis were down-regulated upon anthesis (Figure 4A). This decrease in gene expression was inversely proportional to biomass accumulation, suggesting that glycolysis provides precursors and energy necessary for petal development. Decreased activity upon growth arrest seems to be sufficient to support biosynthesis of secondary metabolites after anthesis. The down-regulation of glycolytic gene expression observed in *A. thaliana* petals upon anthesis [30], along with our results, indicate a common cross-species mechanism for the regulation of glycolysis during petal development. Unlike glycolytic genes, most ESTs in the pentose phosphate pathway did not change over snapdragon petal development (Figure 4B). In contrast, expression of genes involved in the pentose phosphate pathway in *A. thaliana* was down-regulated in petals upon anthesis [30]. This difference between the developmental regulation of the pentose phosphate pathway in *A. thaliana* and snapdragon flowers is likely due to their different volatile compositions. Indeed, *A. thaliana* flowers predominantly emit terpenoid compounds [37], [38], while in snapdragon the phenylpropanoid methylbenzoate is the major volatile, which is ultimately derived from the pentose phosphate pathway intermediate erythrose 4-phosphate.

## Materials and Methods

### Plant Material

Snapdragon (*Antirrhinum majus* cv. Maryland True Pink; Ball Seed, West Chicago, IL) was grown under standard greenhouse conditions as described previously [9]. Upper and lower lobes of petals as well as sepals were harvested and immediately frozen in liquid nitrogen at four stages of flower development: three days before anthesis (d-3), the day of anthesis (d1), four and seven days after anthesis (d4 and d7).

### Quantification of Weight, Total Anthocyanin and Chlorophyll Levels

Snapdragon petals and sepals were collected at 3PM at the four developmental stages described in the results section and their fresh weight immediately determined on a microbalance. The tissue was then desiccated for 18 hours at 90°C and the dry weight measured. Anthocyanins were analyzed as described by [39]. Chlorophyll was measured as described by [40].

### RNA Isolation and Microarray Design

For microarray analysis, the petals and sepals of snapdragon flowers were harvested in triplicates at 11AM from the four different developmental stages. To minimize flower-to-flower and day-to-day variations, at least ten flowers were harvested from three different days and pooled together (thirty flowers total) to generate each biological replicate. Total RNA was isolated from approximately 200 mg of frozen tissues using the RNeasy Plant Mini Kit (Qiagen). A Roche NimbleGen custom 4×72 K expression array was designed based on 12,497 *Antirrhinum majus* unigenes available at National Center for Biotechnology Information (NCBI) and our expression sequence tag (EST) database. Up to six 60-mer probes were designed for each target gene. Preparation of double-stranded cDNAs from the total RNA, labeling of the cDNA with Cy3 dye, and hybridization to the microarray were carried out by MOgene, LC (www.mogene.com). Microarray data were deposited in the NCBI Gene Expression Omnibus database (Accession number GSE36356).

### Extraction and Analysis of Semipolar Metabolites using LC–MS

Frozen powder (100–200 mg) from sepal and petal tissue was extracted with 80% (v/v) MeOH, and the solid:liquid ratio was kept at 1∶3 (w/v). The mixture was sonicated for 20 min at room temperature, vigorously mixed with chloroform and centrifuged (13,000 rpm, 10 min). After phase separation, the aqueous layer was dried using vacuum centrifugation, resuspended in 100 µL of a HPLC diluent of 5% acetonitrile containing 0.1% formic acid and analyzed by LC-MS. Chromatographic separations were performed with an Agilent 1100 HPLC system and an Atlantis T3 (2.1×150 mm×3 um) separation column (Waters, Milford, MA). Mobile phases were (A) 0.1% formic acid (v/v) in LC-MS grade water and (B) 0.1% formic acid (v/v) in acetonitrile. After 5 µL of sample injection, 5% B was held for 1 min, followed by a linear gradient to 75% B over 49 min and a 2 min ramp back to 5% B. Finally, 5% B was equilibrated for 8 min. The flow rate was kept at a 0.3 mL/min and the column temperature at 40°C. The column effluent was then introduced by positive electrospray ionization (ESI) into an Agilent 6210 MSD time-of-flight mass spectrometer. The ESI capillary voltage was 3.0 kV, nitrogen gas temperature was set to 350°C, drying gas flow rate was 11.0 L/min, nebulizer gas pressure was 30 psig, skimmer was 65 V, and OCT RF was 250 V. Mass data (from m/z 70–1500) were collected and analyzed using Agilent MassHunter B.02 software. Data pre-processing was performed using GeneSpring MS (Agilent Technologies).

To tentatively identify peaks detected by LC-TOF-MS, d7 petal extracts were subjected to ion trap LC-MS (Agilent Technologies 1100 Series HPLC system coupled to an Agilent Technologies Mass Selective Trap SL detector). The parameters for liquid chromatography were as described above. The collision energy was set to 1.2eV.

### Analysis of Polar Metabolites, Total Fatty Acids, and Flower Volatiles using GC-MS

Polar metabolites were analyzed as described by [41]. Total fatty acid levels were measured as described by [42]. Volatiles were quantified as described by [10].

### Statistical Analysis

Microarray data were normalized with the RMA method [19]. Non-specific IQR filtering was used to remove probe sets with low variation across the arrays. A one-way ANOVA with the developmental stage as a fixed effect was conducted. The mathematical model used is shown as follows:
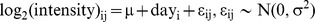



The fixed effect of the day factor was based on zero sum constraint, where ∑ day_i_ = 0 is an identifiability constraint that is required for estimation of model parameters. The random deviation was independent and followed a normal distribution with mean 0 and σ^2^ as the measurement error variance. Three hypotheses were tested: H_0_: µ_d-3_ = µ_d1_, H_0_: µ_d1_ = µ_d4_, H_0_: µ_d4_ = µ_d7_. The false discovery rate was controlled using the Benjamini-Hochberg method [20]. From the results of the three comparisons, a pattern score was calculated. If the comparison was not significantly different, the pattern score 0 was assigned. If the comparison was significantly different and up-regulated, the pattern score was 1. If the comparison was significantly different and down-regulated, the pattern score was −1. Features were grouped into one of the 3^3^ possible score patterns based on the three comparisons. The same procedure was followed for the statistical analysis of pre-processed LC-MS data. All analyses were performed with R and Bioconductor [43].

Clustering of samples based on their gene expression/metabolite abundance profiles was achieved by hierarchical cluster analysis, with Pearson correlation and average linkage serving as correlation and agglomeration methods.

GO term enrichment analysis was performed using the Blast2GO software [21]. Functional annotation of snapdragon ESTs was based on blasting against the *A. thaliana* Genbank protein database. Default parameters were used for the blasting, mapping, and annotation steps.

Correlation analysis between genes and metabolites within different pathways was conducted in Cytoscape [26]. Each EST expression value/metabolite abundance was divided by the average d-3 expression value/metabolite abundance for that EST/metabolite. The ratio was log_2_ transformed. Average log_2_ transformed values/abundances for each stage were used for the correlation analysis. ESTs and metabolites being correlated (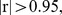 p-value<0.05) to at least one other EST or metabolite were included into the correlation network.

To determine the function of genes that correlate with different metabolite classes, metabolites were first assembled into the following classes: “Terpenoids”, “Phenylalanine, tyrosine, and phenylpropanoids”, “TCA cycle intermediates”, and “Amino acids” (see Table S1 for metabolite class assignment of metabolites). Significance of the Pearson correlation between each metabolite and genes was assessed by a permutation test using R and the permcor function in the permax package (10^4^ permutations). Genes that were significantly correlated (Westfall and Young-corrected p-value <0.05) were assembled into lists. Gene lists were grouped based on the metabolite class they were correlated to. Grouped gene lists were finally used to perform a GO term enrichment analysis using the Blast2GO software [21].

## Supporting Information

Figure S1 **Developmental gene expression in the anthocyanin biosynthetic pathway.** ESTs were annotated and assigned to enzymatic steps based on their homology to A. thaliana genes involved in each enzymatic step. Each EST was assigned a set of three boxes representing the three comparisons made to evaluate changes in gene expression: d-3 vs. d1, d1 vs. d4, d4 vs. d7. The boxes were colored according to the change in gene expression: Red and blue boxes indicate significant up- and down-regulation, respectively, for a given comparison, while white boxes indicate no significant change in gene expression.
(TIF)

Table S1 Assignment of metabolites to metabolite classes
(DOC)

Table S2 Excel file containing peak abundances of petal metabolites measured by LC-MS at different flower developmental stages.
(XLS)

Table S3 Excel file containing peak abundances of sepal metabolites measured by LC-MS at different flower developmental stages.
(XLS)
