# Supplementary figures and images for: Developmental Changes in the Metabolic Network of Snapdragon Flowers
Source: PLoS One. 2012 Jul 11;7(7):e40381. doi: 10.1371/journal.pone.0040381 (PMC3394800; doi:10.1371/journal.pone.0040381)

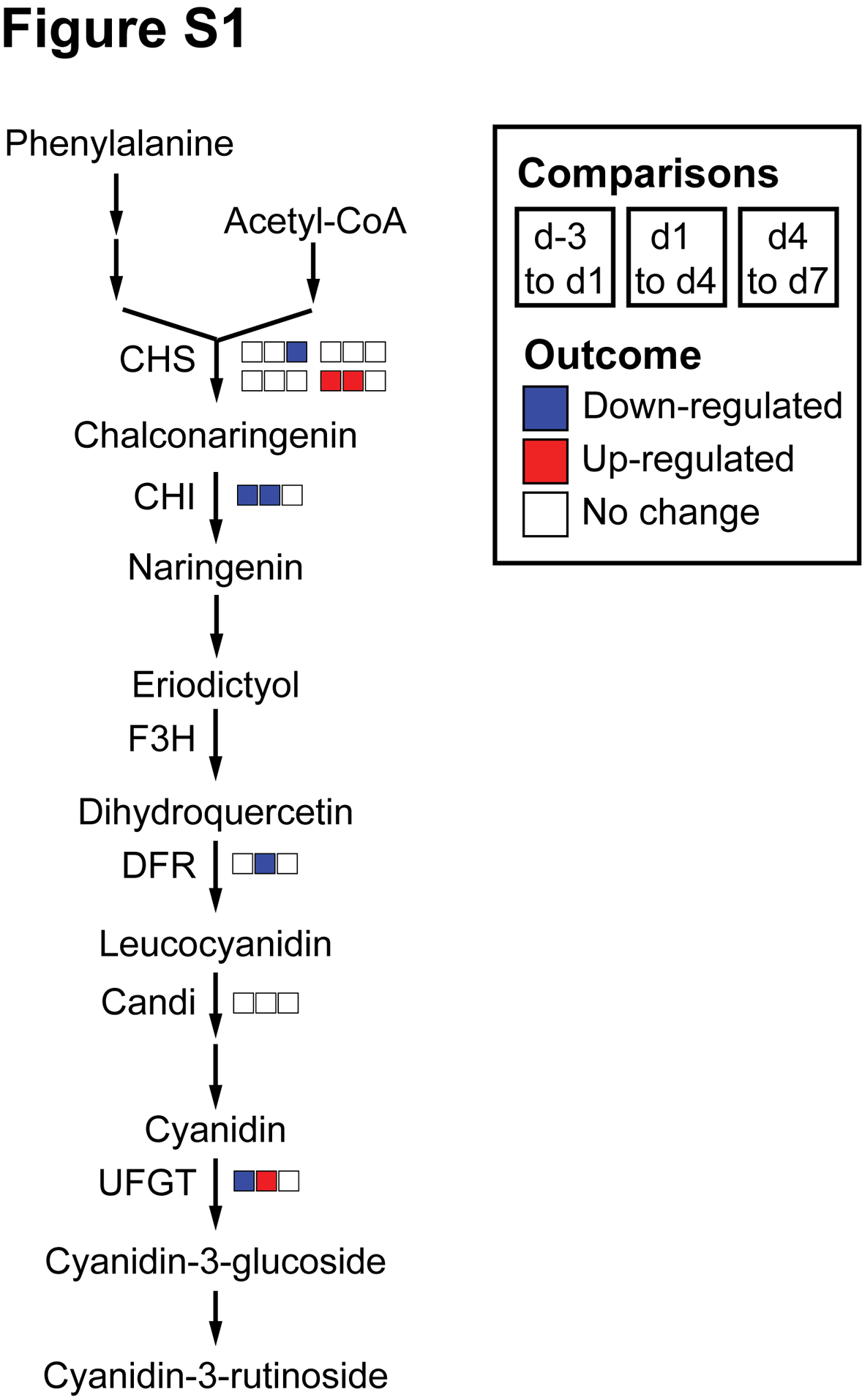

Supplement: Figure S1 — Developmental gene expression in the anthocyanin biosynthetic pathway. ESTs were annotated and assigned to enzymatic steps based on their homology to A. thaliana genes involved in each enzymatic step. Each EST was assigned a set of three boxes representing the three comparisons made to evaluate changes in gene expression: d-3 vs. d1, d1 vs. d4, d4 vs. d7. The boxes were colored according to the change in gene expression: Red and blue boxes indicate significant up- and down-regulation, respectively, for a given comparison, while white boxes indicate no significant change in gene expression. (TIF) [file pone.0040381.s001.tif]
