# Supplementary material for: Developmental Changes in the Metabolic Network of Snapdragon Flowers
Source: PLoS One. 2012 Jul 11;7(7):e40381. doi: 10.1371/journal.pone.0040381 (PMC3394800; doi:10.1371/journal.pone.0040381)
Supplement: Table S1 — Assignment of metabolites to metabolite classes (DOC) [file pone.0040381.s002.doc]

Table S1: Assignment of metabolites to metabolite classes

| **Metabolite** | **Metabolite class** |
| --- | --- |
| Phenylalanine | Phenylalanine, tyrosine, and phenylpropanoids |
| Tyrosine | Phenylalanine, tyrosine, and phenylpropanoids |
| Cinnamic acid | Phenylalanine, tyrosine, and phenylpropanoids |
| Benzoic acid | Phenylalanine, tyrosine, and phenylpropanoids |
| Methylbenzoate | Phenylalanine, tyrosine, and phenylpropanoids |
| 4-hydroxyphenylethanol | Phenylalanine, tyrosine, and phenylpropanoids |
| 4-hydroxybenzoic acid | Phenylalanine, tyrosine, and phenylpropanoids |
| Ocimene | Terpenoids |
| Myrcene | Terpenoids |
| Linalool | Terpenoids |
| Nerolidol | Terpenoids |
| Malic acid | TCA cycle intermediates |
| Fumaric acid | TCA cycle intermediates |
| Succininc acid | TCA cycle intermediates |
| Aspartate | Amino acids |
| Isoleucine | Amino acids |
| Alanine | Amino acids |
| Leucine | Amino acids |
| Valine | Amino acids |
| Threonine | Amino acids |
| Glycine | Amino acids |
| Proline | Amino acids |
| Glutamate | Amino acids |
| Serine | Amino acids |
